# Selective Generation of Aldimine and Ketimine Tautomers of the Schiff Base Condensates of Amino Acids with Imidazole Aldehydes or of Imidazole Methanamines with Pyruvates—Isomeric Control with 2- vs. 4-Substituted Imidazoles

**DOI:** 10.3390/molecules29061324

**Published:** 2024-03-16

**Authors:** Greg Brewer, Cynthia Brewer, Raymond J. Butcher, Peter Zavalij

**Affiliations:** 1 Department of Chemistry, The Catholic University of America, Washington, DC 20064, USA; brewerc@cua.edu; 2 Department of Chemistry, Howard University, Washington, DC 20059, USA; rbutcher99@yahoo.com; 3 Department of Chemistry, University of Maryland, College Park, MD 20742, USA; pzavalij@umd.edu

**Keywords:** imidazole, Schiff base, tautomerization, amino acids, pyruvates, aldimine, ketimine, homochirality

## Abstract

The Schiff base condensation of 5-methyl-4-imidazole carboxaldehyde, 5Me4ImCHO, and the anion of an amino acid, H_2_N-CH(R)CO_2_^−^ (R = -CH_3_, -CH(CH_3_)_2_ and -CH_2_CH(CH_3_)_2_), gives the aldimine tautomer, Im-CH=N-CH(R)CO_2_^−^, while that of 5-methylimidazole-4-methanamine, 5MeIm-4-CH_2_NH_2_, with a 2-oxocarboxylate anion, R-C(O)-CO_2_^−^, gives the isomeric ketimine tautomer, Im-CH_2_-N=C(R)CO_2_^−^. All are isolated as the neutral nickel(II) complexes, NiL_2_, and are characterized by single crystal structure determination, IR, and positive ion ESI MS. In the cases of the 4 substituted imidazoles, either 5MeIm-4-CHO or 5MeIm-4-CH_2_NH_2_, both the aldimine and ketimine complexes are isolated cleanly with no evidence of an equilibrium between the two tautomers under the experimental conditions. The aldimines are blue while the tautomeric ketimines are green. In contrast, for the 2-substituted imidazoles, with either Im-2-CHO or Im-2-CH_2_NH_2_, the isolated product from the Schiff base condensation is the ketimine, which in the solid is green, as observed for the 4-isomer. These results suggest that for the 2-substituted imidazoles, there is a facile equilibrium between the aldimine and ketimine tautomers, and that the ketimine form is the thermodynamically favored tautomer. The aldimine tautomers of the 4-substituted imidazoles have three stereogenic centers, the nickel (Δ or Ʌ) and the two alpha carbon atoms (R or S). The observed pair of enantiomers is the ɅRR/ΔSS enantiomeric pair, suggesting that this pair is lower in energy than the others and that this is in general the preferred chiral correlation in these complexes.

## 1. Introduction

**Figure 1:**
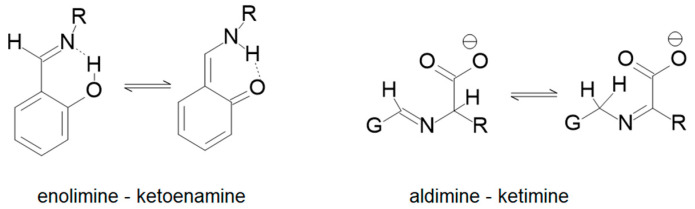
Two types of tautomerism observed in Schiff bases.

Schiff base complexes are widely used in chemistry to study diverse topics including spin crossover [1,2,3,4,5], models of enzyme systems [6,7,8] and supramolecular complexes [9,10] due to their ease of preparation and broad versatility. Two types of tautomerization have been reported for this chemical moiety, enol imine–keto enamine and aldimine–ketimine as shown in Figure 1 [11,12].

**Figure 2:**
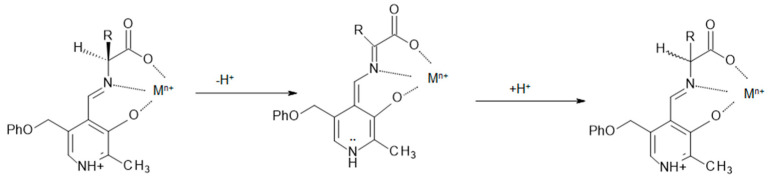
The proposed steps in the racemization of an AA. Deprotonation of the chiral aldimine at C_α_ followed by tautomerization gives an achiral ketimine. Reprotonation of the ketimine gives an aldimine, which has a stereogenic center at C_α_ but is now racemic.

The first has been studied at great length and structurally characterized. So far, it has found relevance in the coordination of various metals, development of organic frameworks, molecular switching, medicinal chemistry and chemosensors [13,14,15,16]. The aldimine–ketimine tautomerization, although it has been observed in solution for decades [17,18,19,20], has eluded attempts to structurally characterize either form as a rearrangement of the other until recently [21]. This type of tautomerism is important to an understanding of the function of pyridoxal in pyridoxal-containing enzymes for the racemization, transamination, decarboxylation, and other enzymatic reactions of amino acids, AA [22,23,24]. Racemization of alanine is a relatively simple example of a mechanism proposed for a pyridoxal-containing enzyme. It is thought that this process involves the tautomerization of an aldimine formed between pyridoxal and alanine to a ketimine form, which can be described as the Schiff base that would be formed between pyridoxamine and pyruvate. The tautomerization of the aldimine to a ketimine involves the loss of the amino acid α proton with the creation of a C_α_ = N_AA_ double bond, putting the C_α_ atom in an achiral environment. Subsequent addition of a proton to C_α_ from another amino acid residue can occur from either side of the C_α_ = N_AA_ double bond, leading to racemization. Recent work proposes this type of mechanism for the racemization and transamination of amino acids by pyridoxal phosphate, PLP, and a metal ion even in the absence of an enzyme [25]. Figure 2 shows this mechanism for the racemization of an amino acid.

An interesting study that verified the existence of this type of tautomerism and produced kinetic data for the conversion of ketimine to aldimine was carried out by Holm and Weinstein [26,27] for salicylaldehyde adducts with amino acids. Along with other model complexes of the adduct of pyridoxal enzymes and amino acids, the Cu^2+^ complex of the ketimine formed between o-hydroxybenzylamine and pyruvic acid was synthesized but not structurally characterized. The base catalyzed tautomerization of this complex to its aldimine form, the Cu^2+^ complex of the Schiff base of salicylaldehyde and alanine, was then followed spectroscopically. The ketimine was chosen as a starting material to follow its conversion to the aldimine in solution because the authors concluded that, under their conditions, the aldimine is more stable than the ketimine. Aldimine solutions contained very little ketimine and the aldimine solutions were easily analyzed for aldimine bands. It was therefore convenient to observe the conversion of ketimine to aldimine as the ketimine absorptions in solution were replaced by aldimine absorptions.

The easy conversion of the ketimine to the aldimine in this system, but not the reverse, is in contrast with the current belief that this tautomerism plays an important role in the functioning of pyridoxal-containing systems—a ready formation of the ketimine from the aldimine in early steps of the mechanisms is proposed for the reactions of PLP-containing enzymes. It is widely accepted that PLP serves as an electron sink in stabilizing the negative charge that develops in intermediates that are generated in reactions of PLP with AA’s. It has been suggested that the nitrogen atom of the pyridoxal ring and its position in the ring is responsible for the stabilization of the intermediate of the conversion of the aldimine form to the ketimine form in these systems. That ring position provides a sink through conjugation for the negative charge that is generated when the α proton of the amino acid fragment is removed. In contrast, Weinstein’s and Holm’s system contained only a simple aromatic moiety with no heteroatom.

Keto–enol tautomerism is similar to the aldimine–ketimine equilibrium proposed for the pyridoxal-containing systems and discussed here. The position of the keto–enol equilibrium is affected by many factors governing the stabilization of the enol form, such as H-bonding, steric bulk of substituents, solvent, and conjugation, all of which affect the enol vs. keto stability. For simple carbonyl compounds, the keto–enol equilibrium strongly favors the keto tautomer. However, this is not always the case, as even vinyl alcohol, the enol tautomer of acetaldehyde, has been observed to be pi-bound to a platinum ion in complexes [28,29]. In these cases, stabilization of the enol form is due to the double bond of the enol providing two electrons to give the platinum atom a stable electron count. Stabilization of the enol form is also observed when conjugation of the enol double bond is possible with another pi system, as observed in many structurally characterized systems [30,31,32,33]. Both the enol and keto forms of the neutral hexafluoroacetylacetone have been structurally characterized [34,35].

For both the basic keto–enol and the aldimine–ketimine tautomerizations (1,3 H shifts), the mechanism of the tautomerization begins with an abstraction of a proton from a carbon atom adjacent to the carbonyl. This leaves a pair of electrons, which provides the impetus for the observed rearrangement. The ability to stabilize the negative charge plays a role in determining whether or not the rearrangement is likely to take place. In the keto/enol case, trifluoroacetaldehyde and hexafluoroacetylacetone are more prone to undergo a keto–enol tautomerization than acetaldehyde and acetylacetone due to the presence of the electronegative fluorine atoms [36,37]. For the aldimine–ketimine tautomerizations reported here, the stabilization of the negative charge is accomplished through resonance delocalization to an electronegative substituent, a nitrogen atom of an imidazole ring.

We report here the structural characterization of the Schiff base condensations of 2- and 4-imidazolecarboxaldehydes with amino acids to produce aldimines, of 2- and 4-substituted methanamine imidazoles with α-keto acids to produce the corresponding ketimines and the isomeric dependence of the tautomerization of aldimines to ketimines. We conclude from this work that there is isomeric, 2- vs. 4-substituted imidazoles, control over the aldimine–ketimine equilibrium. For 2-substituted imidazoles, the ketimine is the observed tautomer and for the 4-substituted imidazoles there is no equilibrium observed, and both aldimine and ketimine products can be produced and isolated. The position of the nitrogen atoms in the imidazole ring, relative to its substituent, determines if the aldimine–ketimine equilibrium is observed or not. As with pyridoxal itself, the 2-substituted imidazoles allow for the stabilization of the intermediate in the formation of the ketimine by providing a sink through conjugation for the negative charge that is generated when the α proton of the amino acid is removed.

## 2. Results and Discussion

### 2.1. Ligands Employed and Reactions Examined

**Figure 3:**
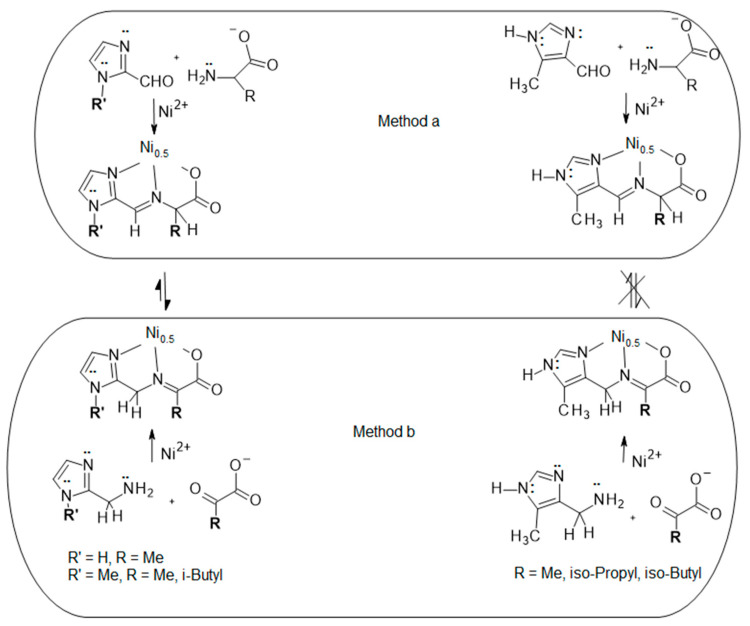
The twelve reactions discussed in this study. Method (a), top, is the reaction of an imidazole carboxaldhyde with the anion of an amino acid to produce an aldimine complex, if there is no tautomerization. Method (b), bottom, is the reaction of a methanamine imidazole with a pyruvate to produce a ketimine complex, if there is no tautomerization. Reactions on the left are of a 2-substituted imidazole (carboxaldehyde or methanamine), while those on the right are of a 4-substituted imidazole (carboxaldehyde or methanamine). For the reactions on the left, R’ = H and R = Me, or R’ = Me and R = Me, or i-Butyl; while for those on the right, R = Me, i-propyl or i-butyl. The reaction conditions for all method (a) reactions are thirty minutes in refluxing methanol. For method (b), the reflux times are extended to three to ten hours. Full details for all reactions are given in Section 3.4.

The ligands utilized and the reactions conducted in this study are pictured in Figure 3. The R groups on the amino acids were restricted to Me, i-propyl and i-butyl, as these contain no donor atoms that could bind to the nickel(II) ion, which could significantly affect the structure of the complexes. The electronic properties of other side chains and their substituent effects have been reported [38]. The Schiff base condensations can be carried out by two alternate methods: (a) reaction of the deprotonated anions of the amino acids alanine (A), valine (V) and leucine (L) with an imidazole carboxaldehyde, or (b) reaction of the sodium salts of pyruvic, 3-methyl-2-oxobutyric or 4-methyl-2-oxovaleric acids with a methanamine imidazole, as shown in Figure 3. The resulting Schiff base, assuming no tautomerization, is an aldimine if prepared by method (a) or a ketimine if prepared by method (b).

**Figure 4:**
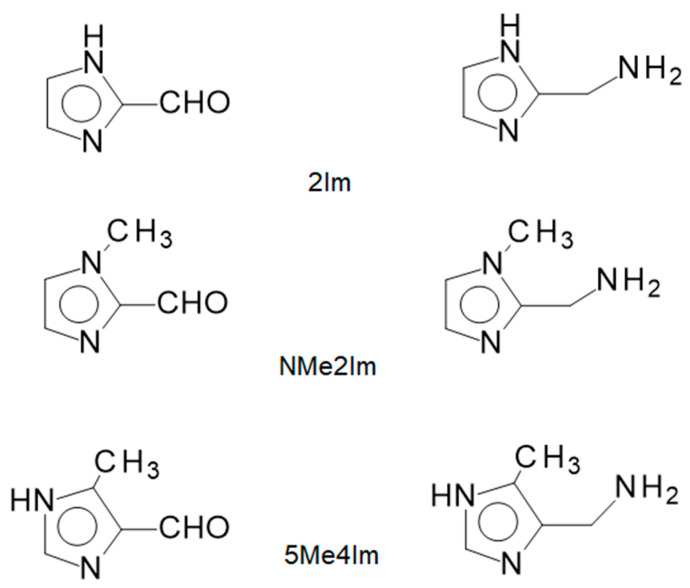
Line drawings giving the abbreviations used for both the aldehydes on the left and the methanamines on the right. In terms of the Schiff base condensations, the aldehydes on left are equivalent to the methanamines on the right when condensed by method (a) and method (b), respectively (Figure 3). In a similar fashion, the amino acid anions of, A, V and L are synthetically equivalent to sodium salts of pyruvic(A), 3-methyl-2-oxobutyric(V) or 4-methyl-2-oxovaleric(L) acid.

In terms of the Schiff base products, an imidazole carboxaldehyde. method (a). and its corresponding methanamineimidazole, method (b), can be regarded as synthetic equivalents as illustrated in Figure 4. In a similar fashion, the amino acid anions of A, V and L method (a) are synthetically equivalent to the sodium salts of pyruvic(A), 3-methyl-2-oxobutyric(V) or 4-methyl-2-oxovaleric(L) acids.

**Figure 5:**
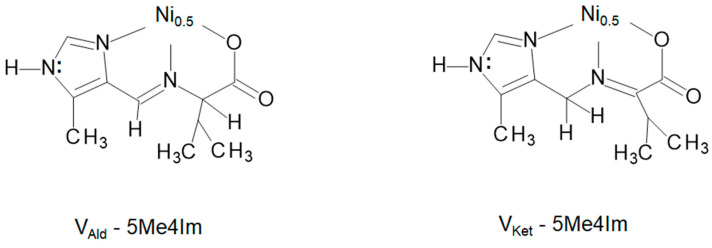
Line drawings illustrating that the difference between Ni(V_Ald_-5Me4Im)_2_ and Ni(V_Ket_-5Me4Im)_2_ is the location of the imine bond. For the aldimine (**left**) the imine bond is between the nitrogen atom of the amino acid, N_AA_, and aldehyde carbon atom, C_ald_, while for the ketimine (**right**) it is between N_AA_ and alpha carbon atom of the amino acid, C_α_.

The ligands are represented as the one letter symbol for the anion of the amino acid (or its corresponding 2-oxocarboxylate) followed by the symbol for the imidazole aldehyde (or its corresponding imidazole methanamine). Both have identical backbones and differ only in the location of the imine bond found from crystallographic analysis. This distinction, aldimine or ketimine, is made by inclusion of Ald or Ket as a subscript following the letter of the amino acid anion (or its corresponding 2-oxocarboxylate) and is illustrated for the tautomers of valine in Figure 5. All the Schiff base condensates are isolated as their neutral nickel(II) complexes, Ni(ligand)_2_.

Six pairs of reactions (12 total reactions), three using a 2-substituted imidazole by both methods (a) and (b) and three using a 4-substituted imidazole by methods (a) and (b) are presented as depicted in Figure 3. There are three distinct outcomes of this study of twelve reactions. (1) If there is no aldimine/ketimine tautomerization under the employed conditions, there would be twelve different products. (2) Six different products would be expected if tautomerism was possible with both the 2- and 4-substituted imidazoles and the more stable tautomer in each case was isolated. (3) Lastly, if tautomerization was observed with one isomeric imidazole but not the other, and the more stable tautomer was isolated, there should be nine products. Outcome 3 is what was found as described below. The crystallographic information for the nine products is in Table S1 in the Supplemental Information.

### 2.2. Methods (a) and (b) for 2Im and NMe2Im

**Figure 6:**
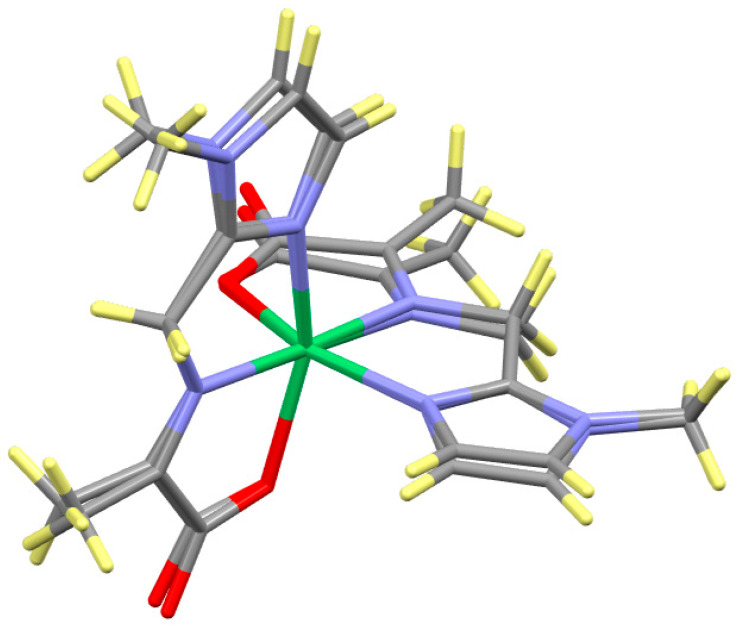
Overlay of structural diagrams of the triclinic Ni(A_ket_-NMe2Im)_2_ trihydrate produced by method (a) and monoclinic Ni(A_ket_-NMe2Im)_2_ dihydrate produced by method (b) illustrating the overall similarity. The overlayed atoms for each complex are the Ni(II) and its six donor atoms. RMS is 0.0799. The colors designate the type of atom, Ni (green), C (gray), N (blue), O (red) and H (yellow).

In our earlier report [21] we presented the results for method (a) for ten different 2-substitued imidazole carboxaldehydes. Unexpectedly, the products were all ketimines rather than the anticipated aldimines. Three of these products, Ni(A_ket_-2Im)_2_, Ni(A_ket_-NMe2Im)_2_ and Ni(L_ket_-NMe2Im)_2_ were also prepared by method (b), as described in the Experimental section and structurally characterized in this study to determine if these were identical to those produced by method (a). The Ni(A_ket_-2Im)_2_ and Ni(L_ket_-NMe2Im)_2_ produced by method (b) in this study were identical to those made by method (a), as they crystallized in the same space groups with identical cells as those prepared by method (a). When prepared by method (b), Ni(A_ket_-NMe2Im)_2_ crystallized as a monoclinic dihydrate rather than a triclinic trihydrate when prepared by method (a). The location of the double bond (ketimine tautomer) was identical in both cases. There is no significant structural difference between the two polymorphs of Ni(A_ket_-NMe2Im)_2_ produced by methods (a) and (b) as is shown in Figure 6.

This result shows that identical 2-substituted imidazoles are produced by both methods (a) and (b) in the three cases examined and presented here. Reactions performed by method (a) to give the aldimine produce only ketimine by a 1,3 hydrogen shift and those performed by method (b) give only ketimine. This supports the conclusion that tautomerization between aldimine and ketimine is observed in these cases as reactions performed by method (a) do not generate the anticipated aldimines but instead give the ketimines, which are identical to the products produced from method (b). A method (a) reaction mixture must contain both tautomers at some ratio as initially the aldimine is formed and then tautomerizes to the ketimine, which is the only observed product. A method has been reported for the determination of tautomer ratios in solution from free energy data [39], but this was not performed for these reactions due to the lack of needed data, rigor, complexity and cost of these methods. A method (b) reaction mixture, which produces the ketimine, may or may not contain some aldimine, but only ketimine is observed for the 2Im and NMe2Im reactions performed by either methods (a) or (b). A solution containing an equilibrium mixture of aldimine (90%) and ketimine (10%) may deposit only ketimine solid if it was the significantly less soluble product, due to two simultaneous equilibria, tautomerization and crystallization. Since the ketimine tautomer is the only product isolated, it is the thermodynamic product, the less soluble product, or both. We suggest that the ketimine is the more stable tautomer as there is no structural difference between these tautomers, other than the location of the imine bond, that suggests a solubility difference.

### 2.3. Methods (a) and (b) for 5Me4Im

**Figure 7:**
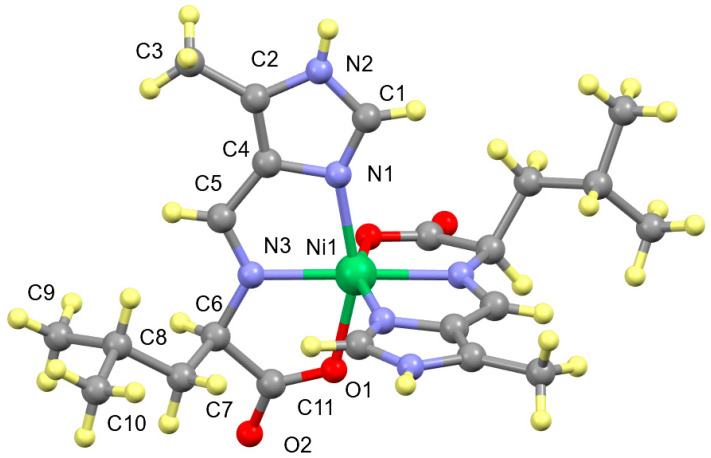
Structure of Ni(L_Ald_-5Me4Im)_2_, which is representative of Ni(A_Ald_-5Me4Im)_2_, Ni(A_Ket_-5Me4Im)_2_, Ni(V_Ald_-5Me4Im)_2_, Ni(V_Ket_-5Me4Im)_2_ and Ni(L_Ket_-5Me4Im)_2_, ignoring the location of the imine bond and the alkyl substituents. The colors designate the type of atom, Ni (green), C (gray), N (blue), O (red) and H (yellow).

The six reactions depicted on the right hand side of Figure 3 were performed as described in the Experimental section. Six products were isolated, three aldimines prepared by method (a) and three ketimines prepared by method (b). The coordination geometries of these six octahedral complexes are very similar to those described earlier [21] and to one another. The structure of Ni(L_Ald_-5Me4Im)_2_ is given in Figure 7 and is representative of all six of these complexes, neglecting the location of the imine bond and the alkyl substituent (Me, i-propyl or i-butyl).

**Figure 8:**
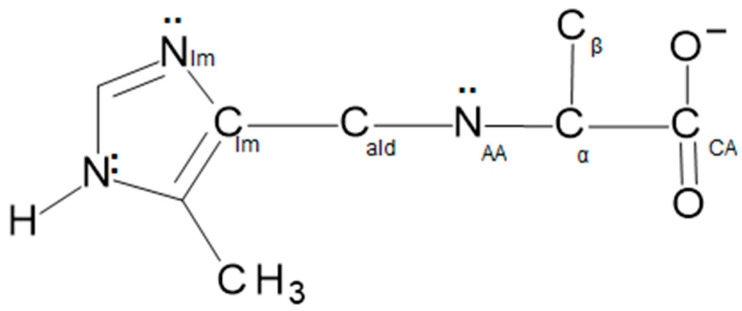
Line drawing illustrating the connectivity in the aldimine and ketimine tautomers of the Schiff bases prepared from 5Me4Im by methods (a) and (b). The difference between the tautomers is the location of the imine bond. The double bond for the aldimine is between N_AA_ and C_ald_, and for the ketimine it is between N_AA_ and C_α_. The ligands bind to the nickel(II) ion through the N_Im_, N_AA_ and O atoms.

The two tridentate ligands bind meridionally to the nickel(II) ion through the imidazole nitrogen, N_Im_, the amino acid nitrogen, N_AA_, and carboxylate oxygen, O, atoms (see Figure 8). The steric bulk of the side chains has no effect on the structure of the complexes, as they are on opposite sides of the molecule. The two ligands are roughly perpendicular to one another with a trans N_AA_–Ni-N_AA’_ angle. The other two trans angles are the two N_Im_–Ni-O angles of each ligand. The distortions from octahedral geometry are because there are only seven atoms, N_Im_, C_Im_, C_Ald_, N_AA_, C_α_, C_CA_ and O, in the backbone of the fairly rigid ligand, which links trans positions on the nickel(II). All the Ni-donor atom distances are in the range of ~2.05–2.10 Å. There is no significant difference between the coordination geometries of the aldimine and ketimine complexes as shown in Supplementary Materials Table S2.

In contrast to the lack of structural differences between the aldimine and ketimine complexes when examining their coordination geometry, there are significant differences between these forms when C_ald_, N_AA_ and C_α_ are examined. This is because C_ald_ and C_α_ are three- and four-coordinate, respectively, in the aldimine form but four- and three-coordinate, respectively, in the ketimine form. This is due to the 1,3 movement of the C_α_ hydrogen atom from C_α_ to C_ald_. These significant changes in bond number (3 vs. 4), bond distances and bond angles are expected when the hybridization changes as it does for C_ald_ and C_α_. The C_ald_ to N_AA_ bond distance increases from ~1.27 to 1.46 Å, while the C_α_ to N_AA_ bond distance decreases from ~1.46 to 1.27 Å on switching from the aldimine to the ketimine tautomer. The change in average bond angles of C_ald_ and C_α_ are from ~120 to ~109°, and the reverse, respectively, for the two tautomers. There is no change in hybridization for N_AA_, so its bond angles undergo little change between the tautomers. Table 1 documents all the metric changes between the aldimine and ketimine forms of the Schiff base condensates of A with 5Me4Im for C_ald_, N_AA_, and C_α_. Table S3 in the Supplementary Materials, provide the same metrics for (b) Ni(V_Ald_-5Me4Im)_2_ and Ni(V_Ket_-5Me4Im)_2_ and (c) R=iBu Ni(L_Ald_-5Me4Im)_2_ and Ni(L_Ket_-5Me4Im)_2_.

**Figure 9:**
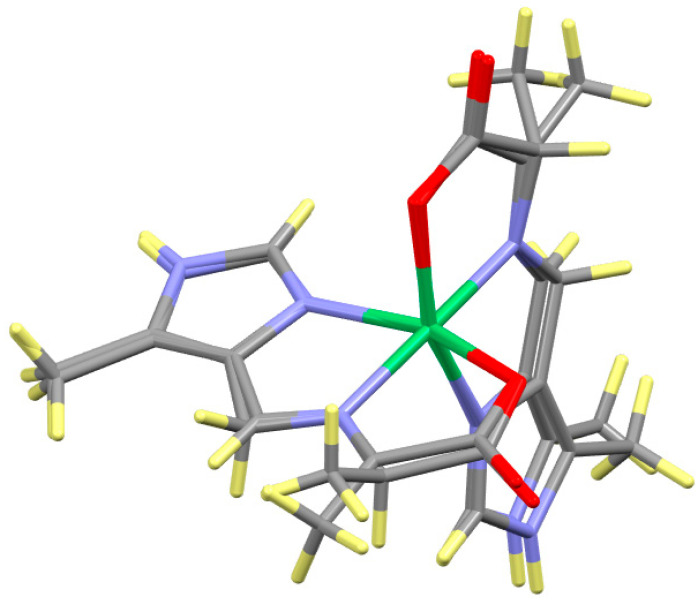
The overlay of the Ni(A_Ald_-5Me4Im)_2_ and Ni(A_Ket_-5Me4Im)_2_ structures depicting both the overall similarities and the significant differences at C_ald_ and C_α_. The overlayed atoms are the Ni(II) and its six donor atoms of each complex. RMS = 0.0571. Note C_ald_ and C_α_. (lower left and upper right). The lone H atom on the C_ald_ of the aldimine form bisects the geminal H atoms of the ketimine form, and the Me group on the C_α_ of the ketimine form bisects the H atom and the Me group on the same atom of the aldimine form. The colors designate the type of atom, Ni (green), C (gray), N (blue), O (red) and H (yellow).

The differences, given in Table 1, are seen with an overlay of an aldimine and ketimine complex as shown in Figure 9. This reveals overall great similarity but with significant differences at C_Ald_ and C_α_, and little difference at N_AA_, as its CN was unchanged.

The conclusion is that there is currently no evidence of equilibrium under the conditions employed for the six reactions performed with 5Me4Im from the right hand side of Figure 3. A method (a) reaction yields only aldimine and a method (b) reaction yields only ketimine. If there were an equilibrium for the 5Me4Im complexes, under the conditions used, then reactions performed by methods (a) and (b) should have yielded the same product (either aldimine or ketimine), or at least a reaction should have yielded both aldimine and ketimine products in different amounts. Since there is no evidence of an equilibrium, no conclusion can be made regarding the identity of the thermodynamic product, aldimine or ketimine, for reactions of 5Me4Im. This contrasts significantly from the reactions of 2Im or NMe2Im discussed in Section 2.2. Predicting tautomer ratios in solution [39], as mentioned in Section 2.2, is not possible for reactions of 5Me4Im, as there is no evidence of an equilibrium.

### 2.4. Explanation for Reactivity Difference between the 2- and 4-Substituted Imidazoles

Clearly there is a significant difference between the Schiff bases formed from 2-substituted imidazoles and 4-substituted imidazoles. All ten complexes reported previously from 2-substituted imidazoles, prepared as aldimines by method (a), rearranged to give ketimines. In addition, three complexes reported here were prepared as ketimines by method (b) and underwent no rearrangement. In contrast, the six 4-substituted imidazole complexes prepared here, three as aldimines by method (a) and three as ketimines by method (b), were isolated as aldimines and ketimines, respectively, with no rearrangement observed. In addition to these, there are other structurally reported Schiff base complexes of an imidazole-4-carboxaldehyde condensed with an amino acid by method (a) that give the anticipated aldimine product with copper(II) [40,41], nickel(II) [42], zinc(II) [43], and several lanthanide(III) ions [44,45]. No tautomerization was observed in these reactions. It is unlikely that reaction conditions can explain this isomeric difference in reactivity since the reaction conditions of both method (a) and (b) were identical for the 2- and 4-substituted imidazoles.

**Figure 10:**
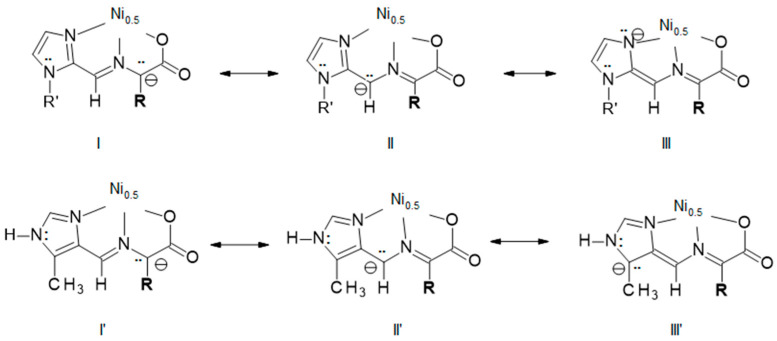
The three resonance structures of the deprotonated aldimine complex of a 2-substituted imidazole (I–III top, R’ is H or Me) and 5Me4Im (I’–III’ bottom). There is a significant stability difference between III and III’, which provides an explanation for the observed isomeric difference in reactivity between 2- and 4-substituted imidazoles.

The racemization of amino acids in the presence of aldehydes (especially pyridoxal), metal ions and a base is well known in the literature and may provide a clue to the reactivity difference. As given in the Introduction (Figure 2), the likely mechanism is the Schiff base condensation of a chiral amino acid with an aldehyde to give the aldimine tautomer with retention of chirality. Deprotonation of C_α_ gives a carbanion which can rearrange to the achiral ketimine. Reprotonation at C_α_ from either side of the double bond regenerates the aldimine, which on hydrolysis gives racemic amino acid. The analogous intermediate carbanion discussed above for the present system (imidazoles) and its resonance structures are shown in Figure 10 for both the 2- and 4-substituted imidazoles. Resonance structures I and I’ and II and II’ are comparable and do not suggest a stability difference. However, there is a significant difference in III and III’ that does provide a possible explanation for the observed isomeric reactivity difference between the 2- and 4-substituted imidazoles. Structure III places a negative charge on an electronegative nitrogen atom. In addition, that nitrogen atom is bound to the nickel(II) ion, which would greatly stabilize the charge. The analogous III’ places the negative charge on a less electronegative carbon atom, and further, that carbon atom cannot have the charge stabilized by binding to the nickel(II) ion.

The long known ability of pyridoxal to racemize amino acids has been attributed to the ability of the nitrogen atom of the pyridine ring to stabilize charge based on its ring position. The argument here is similar: favorable charge stabilization of an imidazole-2-carboxaldehyde intermediate over that of an analogous imidazole-4-carboxaldehyde intermediate may explain the reactivity difference. Resonance structures III and III’ provide a possible explanation for the isomeric dependency that is experimentally observed: imidazole-4-carboxaldehydes give aldimines when reacted with an amino acid anion, but imidazole-2-carboxaldehydes give ketimines under the same conditions.

### 2.5. Chirality Correlation

**Figure 11:**
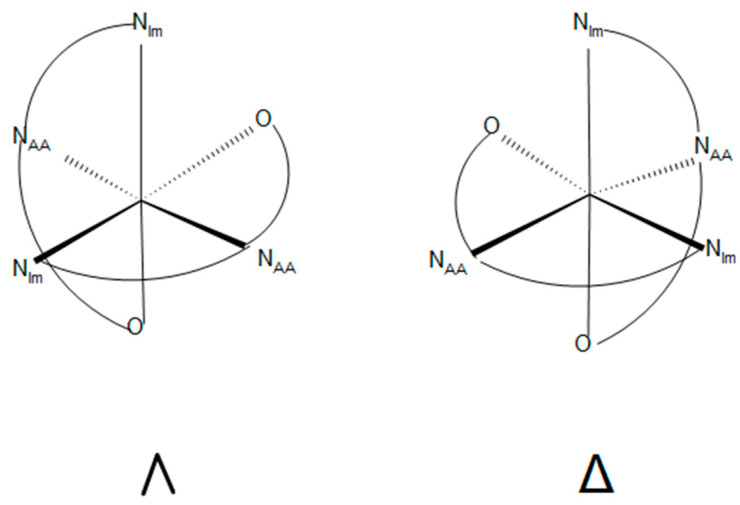
Illustration of the forms of the Λ and Δ aldimine complexes discussed here.

The aldimine complexes have three stereogenic centers, the nickel(II) ion, Δ or Λ as illustrated in Figure 11, and the two alpha carbon atoms, R or S. The designation of Δ or Λ for the meridionally coordinated ligands used here are illustrated below and based on the earlier literature assignments [46].

Three stereogenic centers gives a maximum of eight stereoisomers, four pairs of enantiomers. These are ΔRR/ΛSS, ΔRS/ΛSR, ΔSR/ΛRS and ΛRR/ΔSS. Since there is no distinction between the order of the two alpha carbon centers, one of the ΔSR/ΛRS and ΔRS/ΛSR enantiomeric pairs is redundant and must be deleted leaving three unique pairs of enantiomers, ΔRR/ΛSS, ΔRS/ΛSR and ΛRR/ΔSS. These pairs are diastereomers of one another, and therefore differ in energy due primarily to the bulk of the alkyl group on C_α_. The synthesis of an aldimine complex may result in the isolation of a single pair of enantiomers if the energy difference between the diasteromers is great enough, due to sufficiently large alkyl groups. If the complex crystallizes in a Sohncke space group, then spontaneous enantiomeric separation, at the level of the crystal, but not the bulk, is achieved. Correlation of chirality must be examined for a carefully selected and homologous grouping of molecules, as was performed in an earlier study of reduced Mntren(Im)_3_^2+^ complexes [47].

Table 2 shows the chirality correlation data for nickel(II) aldimine complexes of the Schiff base condensate of an amino acid with an imidazole carboxaldehyde from this study and other available examples, Ni(F_Ald_-5Me4Im)_2_ [42], Ni(F_Ald_-4Im)_2_ [48], both enantiomers of Ni(V_Ald_-2Im)_2_ [49,50] and Ni(L_ket_-NMe2Im)(L_Ald_-NMe2Im) [21]. The observation is that the ΔSS/ΛRR is the only enantiomeric pair observed indicating that it is the pair of lowest energy. Ni(F_Ald_-5Me4Im)_2_ (prepared from L-phenylalanine) crystallized in a Sohncke space group and it is chiral both at the crystal and bulk levels. Surprisingly, both Ni(F_Ald_-4Im)_2_ and Ni(V_Ald_-2Im)_2_ (both prepared from racemic amino acid) crystallize in Sohncke space groups that are enantiomorphic, P4_1_2_1_2 and P4_3_2_1_2. This means that one enantiomer can crystallize in one of the two groups and the second enantiomer must crystallize in the other. In this case the ΔSS molecule crystallizes in P4_3_2_1_2 and the ΛRR in P4_1_2_1_2. These samples are chiral at the level of the crystal, meaning that all the molecules in one crystal are of a single enantiomer. The bulk is still racemic as there would be equal numbers of crystals of opposite chirality.

It is important to consider the observed correlation of chirality in these aldimine complexes in terms of the homochirality of amino acids, essentially all L in nature. Possible explanations for the production of homochiral amino acids from racemic prebiotic sources are significant areas of research [18,19,20], and these results may indicate a possible pathway. The ketimine to aldimine tautomerization creates a stereogenic center at C_α_ and could play a role in the homochirality of amino acids. A metal complex of a ketimine is chiral due to the metal stereogenic center, so the transfer of a hydrogen atom from C_Ald_ to C_α_ may be impacted by the observed correlation of chirality seen in Table 2. A stereoselective or stereospecific hydrogen transfer in a ketimine to an aldimine tautomerization may provide a pathway for enantiomeric enrichment.

### 2.6. Supramolecular Structure

**Figure 12:**
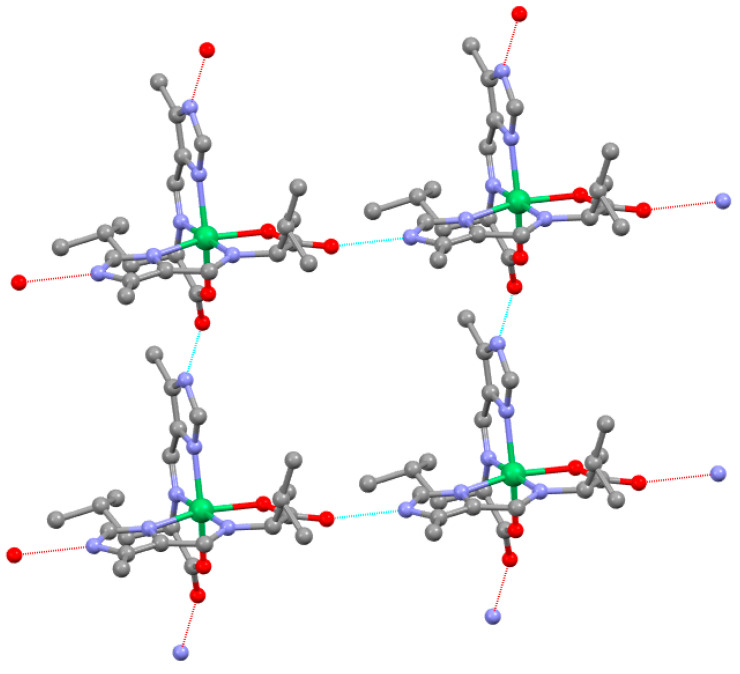
The supramolecular structure of Ni(L_Ald_-5Me4Im)_2_. Hydrogen atoms are omitted for clarity. All complexes of this grid are Λ and all the C_α_‘s are R. Ni(L_Ket_-5Me4Im)_2_ Ni(V_Ald_-5Me4Im)_2_ also exhibit homochiral grids. Ni(V_Ket_-5Me4Im)_2_ gives an achiral grid. The hydrogen bond that holds this together is the N-H ^….^ O (non-nickel bound carboxylate). The N ^….^ O distances are as follows: Ni(V_Ald_-5Me4Im)_2_: 2.629(8), 2.646(9), 2.638(10) and 2.619(9) Å, Ni(V_Ket_-5Me4Im)_2_: 3.0735(19) and 3.023(2) Å. The hydrogen bond in this case is to both carboxylate O atoms, Ni(L_Ald_-5Me4Im)_2_: 2.617(4) Å and Ni(L_Ket_-5Me4Im)_2_: 2.676 Å. The colors designate the type of atom, Ni (green), C (gray), N (blue) and O (red).

The Schiff base ligands prepared here (both aldimine and ketimine forms) have both a hydrogen-bonding donor site, N-H of the imidazole, and an acceptor site, the carboxylate oxygen atom. These sites are at opposite ends of the ligand. In the complexes, two approximately planar ligands are bound to the nickel(II) in a perpendicular arrangement. This sets the stage for interesting supramolecular structures, as the hydrogen donors and acceptors for one molecule are not positioned for intramolecular hydrogen bonding but are oriented for intermolecular hydrogen bonding. Each complex has a ligand that provides a hydrogen donor and acceptor to the left and right, and the other ligand provides a donor and acceptor above and below. Ni(V_Ald_-5Me4Im)_2_, Ni(V_Ket_-5Me4Im)_2_, Ni(L_Ald_-5Me4Im)_2_ and Ni(L_Ket_-5Me4Im)_2_ all crystallize as anhydrous complexes and exhibit the same supramolecular structure as shown in Figure 12. The 2 × 2 grid is held together primarily by the hydrogen bond of an imidazole N-H of one complex to a non-nickel-bound carboxylate oxygen atom of an adjacent complex. As each complex has two donors and two acceptors, the grid is extended throughout the lattice. An interesting observation is that the complexes of a grid arrangement for Ni(V_Ald_-5Me4Im)_2_, Ni(L_Ald_-5Me4Im)_2_ and Ni(L_Ket_-5Me4Im)_2_ are all of the same chirality, either all Δ or all Λ. Given the chirality correlation discussed in the previous section, this means that for Ni(V_Ald_-5Me4Im)_2_ and Ni(L_Ald_-5Me4Im)_2_, the chirality of all of the C_α_ atoms for a Δ grid are S and those for the Λ grid are R. An extended grid is homochiral. However, these complexes crystallize in a non-Sohncke space group, so the crystal is racemic, as the enantiomeric grid is present as well. Ni(V_Ket_-5Me4Im)_2_ exhibits the same grid structure, but the grid is achiral as the four-molecule building block consists of two Δ and two Λ molecules. The extended intermolecular hydrogen bonding may explain the insolubility of these complexes, which rules out solution characterization or studies. Ni(A_Aldt_-5Me4Im)_2_ and Ni(A_Ket_-5Me4Im)_2_ could exhibit this same type of structure, but they crystallize as hydrates, which blocks this extended supramolecular feature.

### 2.7. Visible Spectroscopy of NiN_4_O_2_ Complexes

**Figure 13:**
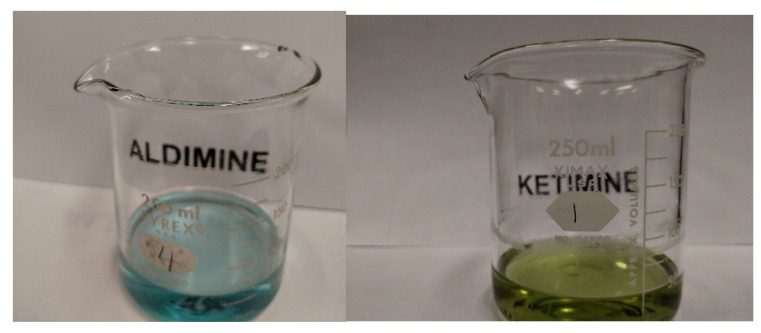
Photographs of the blue Aldimine, Ni(A_Ald_-5Me4Im)_2_ (**left**), and green Ketimine, Ni(A_Ket_-5Me4Im)_2_ (**right**). The photograph underscores that a significant difference in color may be due to small differences in λ_MAX_, 905 and 579 nm vs. 937 and 594 nm. All the aldimine and ketimine complexes of 5Me4Im are blue and green, respectively, with no evidence of tautomerization.

An octahedral nickel(II) complex has three broad d → d transitions, ^3^A → ^3^T_2g_, ^3^A → ^3^T_1g_ and ^3^A → ^3^T_1g_(P), in order of decreasing wavelength, with Δ_Oh_ equal in energy to that of the first transition. The highest energy transition may be obscured if there is a strong peak due to a ligand band. The transitions of Ni(ImH)_6_^2+^ (blue, λ_MAX_: 901, 551 and 348 nm) and Ni(H_2_O)_6_^2+^ (green, λ_MAX_: 1110, 667 and 370 nm) illustrate this and are consistent with their N_6_ and O_6_ donor sets. All the complexes produced here contain the N_4_O_2_ donor set and, the anticipated λ_MAX_ values should be in between those given above for Ni(ImH)_6_^2+^ and Ni(H_2_O)_6_^2+^ with colors in the blue–green region as has been observed for other NiN_4_O_2_ complexes. The observed λ_MAX_ values for Ni(A_Ald_-5Me4Im)_2_ (blue) and Ni(A_Ket_-5Me4Im)_2_ (green) are 905 and 579 nm, and 937 and 594 nm, respectively. In both cases, the shortest wavelength peak is not observed due to the presence of a very intense band below 450 nm, likely associated with the imidazole aldehyde [51,52]. The difference in the λ_MAX_ values between these two complexes is small but suggests that the aldimine form of the ligand is a stronger donor, consistent with its color. The distinction between the aldimine and ketimine complexes is best illustrated in Figure 13. Unfortunately, Ni(V_Ald_-5Me4Im)_2_, Ni(L_Ald_-5Me4Im)_2_, Ni(V_Ket_-5Me4Im)_2_ and Ni(L_Ket_-5Me4Im)_2_ are all insoluble due to their supramolecular structures (see Section 2.6), so solution characterization is not possible. However, both aldimine complexes are blue and the ketimine complexes green in the solid state and in their reaction mixtures.

## 3. Experimental

### 3.1. General

DL Alanine, DL valine, DL leucine, 5-methylimidazole-4-carboxaldehyde, 2-aminomethylimidazole dihydrochloride, 1-(4-methyl-1H-imidazol-5-yl)methanamine hydrochloride, sodium pyruvate, sodium 3-methyl-2-oxobutyrate, sodium 4-methyl-2-oxovalerate, nickel(II) acetate tetrahydrate, methanol and 0.10 M potassium hydroxide in methanol were obtained from Aldrich (St. Louis, MO, USA). 1-Methyl-1H-imidazol-2-yl)methanamine was obtained from Advanced Chem Block (Hayward, CA, USA). All solvents were of reagent grade and used without further purification. IR and UV-visible spectra were obtained on a Perkin Elmer (Waltham, MA, USA) Spectrum Two FT IR and Lambda 365 spectrometer.

### 3.2. ESI-MS Were Obtained by Axis Pharm Laboratory, San Diego, CA

The positive ion MS of all samples were determined as methanol solutions or slurries.

### 3.3. X-ray Crystallography

Crystal data for all complexes were collected on a Bruker (Billerica, MA, USA) Smart Apex II 1000 CCD or on an Oxford Gemini diffractometer. All structures were solved using the direct methods program SHELXS-97 [53]. All nonsolvent heavy atoms were located using subsequent difference Fourier syntheses. The structures were refined against F^2^ with the program SHELXL [54,55], in which all data collected were used, including negative intensities. All nonsolvent heavy atoms were refined anisotropically. All hydrogen atoms were located by Fourier difference. Complete crystallographic details are given in Table S1.

### 3.4. Syntheses

Ni(A_ket_-2Im)_2_: 2-Aminomethylimidazole dihydrochloride (0.170 g, 1.0 mmol) was added to a round bottom flask. KOH was added (20 mL of 0.1 M KOH in methanol, 2 mmols) to deprotonate the hydrochloride salt. Sodium pyruvate (0.110 g. 1.0 mmol) and methanol (10 mL) were added to the flask and the reaction mixture was refluxed for two hours. Ni(acetate)_2_ was added (10 mL of 0.05 M Ni(acetate)_2_ in methanol, 0.5 mmol). The solution was set aside to concentrate. After several days, a crystalline solid was deposited (0.063 g, 32%).

Ni(A_ket_–NMe2Im)_2_: 1-Methyl-1H-imidazol-2-yl)methanamine (0.225 g, 2 mmols) was weighed into a round bottom flask. Methanol (60 mL) and sodium pyruvate (0.220 g, 2 mmols) were added. The reaction mixture was refluxed for 3.5 h. The reaction mixture was removed from reflux and Ni(acetate)_2_ was added (20 mL of 0.05 M Ni(acetate)_2_ in methanol, 1 mmol). The clear, lime-green solution was set aside to concentrate. After two days, large dark crystals of the product had formed (0.289 g, 69%).

Ni(L_ket_–NMe2Im)_2_: 1-Methyl-1H-imidazol-2-yl)methanamine (0.227 g. 2.0 mmols) was weighed into a round bottom flask. Sodium 4-methyl-2-oxovalerate (0.307 g, 2.0 mmols) was added along with 60 mL of methanol. The reaction mixture was refluxed for 10 h. The reaction mixture was removed from reflux and Ni(acetate)_2_ (20 mL of 0.05 M Ni(acetate)_2_ in methanol, 1 mmol) was added. The reaction mixture turned green and was set aside to concentrate. After several days, it was filtered to give a green crystalline product (0.312 g, 62%).

Ni(A_ald_–5Me4Im): D,L-Alanine (0.089 g, 1.0 mmol) was added to 5 mL of water in a round bottom flask, and KOH was added (10 mL of 0.1 M KOH in methanol, 1.0 mmol). Then 5-Methylimidazole-4-carboxaldehyde (0.110 g, 1.0 mmol) and 15 mL of methanol were added. The reaction mixture was refluxed for 30–40 min. Ni(acetate)_2_ was added (10 mL of 0.05 M Ni(acetate)_2_ in methanol, 0.5 mmol). The reaction mixture turned blue. The solution was set aside to concentrate. After several days, a solid was deposited (0.246 g, 59%). The visible bands of Ni(A_Ald_-5Me4Im)_2_ (blue) are 905 and 579 nm.

Ni(V_ald_-5Me4Im)_2_: The same procedure was followed for the reaction of D,L-valine (0.117 g, 1.0 mmol) and 5-methylimidazole-4-carboxaldehyde(0.110 g, 1.0 mmol). Blue crystals formed after several days (0.165 g, 70%).

Ni(L_ald_-5Me4Im)_2_: The same procedure was followed for the reaction of leucine (0.132 g 1.0 mmol) and 5-methylimidazole-4-carboxaldehyde (0.111 g, 1.0 mmol). After several days, a blue crystalline product was obtained (0.146 g, 58%).

Ni(A_ket_–5Me4Im)_2_**:** 1-(4-Methyl-1H-imidazol-5-yl)methanamine hydrochloride (0.199 g, 1.35 mmol) was weighed into a round bottom flask. KOH (13.5 mL of 0.1 MKOH in methanol, 1.35 mmol) was added to deprotonate the hydrochloride salt. Sodium pyruvate (0.147 g, 1.35 mmol) and methanol (27 mL) were added. The reaction mixture was refluxed for ten hours. Ni(acetate)_2_ (13.5 mL of 0.05 M Ni(acetate)_2_ in methanol, 0.675 mmols) was added to give a green solution. The reaction mixture was set aside to concentrate. The reaction mixture went dry without producing crystals. The reaction mixture was redissolved and filtered to remove some whitish, methanol insoluble material. The filtrate was set aside to concentrate and produced solid (0.183 g, 65%) after several days. The observed visible bands for Ni(A_Ket_-5Me4Im)_2_ (green) are 937 and 594 nm.

Ni(V_ket_–5Me4Im)_2_**:** 1-(4-Methyl-1H-imidazol-5-yl)methanamine hydrochloride (0.295 g, 2.0 mmols) was added to a round bottom flask. KOH was added (20 mL of 0.1 M KOH in methanol, 2 mmols) to deprotonate the hydrochloride salt. Sodium 3-Methyl-2-oxobutyrate (0.296 g, 2.0 mmols) and methanol (60 mL) were added. The reaction mixture was refluxed for 10 h. The reaction mixture was removed from reflux and Ni(acetate)_2_ (20 mL of 0.05 M Ni(acetate)_2_ in methanol, 1.0 mmol) was added to give a green solution. The solution was set aside to concentrate. It produced a mixture of green and white solid. The mixture was cleaned up by filtering twice with methanol and the filtrate was set aside. After several days it produced a small amount of a green–brown crystalline solid (0.034 g, 7.2%).

Ni(L_ket_–5Me4Im)_2_: 1-(4-Methyl-1H-imidazol-5-yl)methanamine hydrochloride (0.100 g, 0.68 mmols) was weighed into a round bottom flask. KOH (6.8 mL of 0.1 M KOH in methanol, 0.68 mmols) was added to deprotonate the hydrochloride salt. Sodium 4-methyl-2-oxovalerate (0.103 g, 0.68 mmol) and methanol (33.2 mL) were added. The reaction mixture was refluxed. At the end of 10 h, the reaction mixture was removed from reflux and Ni(acetate)_2_ was added (6.8 mL of 0.05 M Ni(acetate)_2_ in methanol, 0.34 mmols). The green reaction mixture was set aside to concentrate. After several days, the reaction mixture was filtered to give a green crystalline solid (0.046 g, 27%).

### 3.5. Available Data

The following is a list of nickel complexes that provides their CCDC deposition numbers (all structural information is available free of charge from the Cambridge Structural Database) and their observed m/e values in the molecular ion region in the positive ion mode ESIMS to nearest mass. The IR and positive ion ESI MS spectra of each complex are included as Supplementary Materials.

Ni(A_Ket_-2Im)_2_: 2015580, [NiL_2_H^+^] = 391, [NiL_2_Na^+^] = 413, [NiL_2_K^+^] = 429, [Ni_2_L_3_^+^] = 614, [Ni_2_L_4_Na^+^] = 803 and [Ni_2_L_4_K^+^] = 819.

Ni(A_Ket_-NMe2Im)_2_: 2285095, [NiL_2_H^+^] = 419, [NiL_2_Na^+^] = 441, [NiL_2_K^+^] = 457, [Ni_2_L_3_^+^] = 656, [Ni_2_L_4_Na^+^] = 859 and [Ni_2_L_4_K^+^] = 875

Ni(L_Ket_-NMe2Im)_2_: 2323328, [NiL_2_-ipropyl^+^] = 459 [NiL_2_H^+^] = 503, [NiL_2_Na^+^] = 525, [Ni_2_L_3_^+^] = 782,

Ni(A_Ald_-5Me4Im)_2_: 2285097, [NiL_2_H^+^] = 419, [NiL_2_Na^+^] = 441, [NiL_2_K^+^] = 457, [Ni_2_L_3_^+^] = 656

Ni(V_Ald_-5Me4Im)_2_: 2206541, [NiL_2_H^+^] = 475, [NiL_2_Na^+^] = 497, [NiL_2_K^+^] = 513, [Ni_2_L_3_^+^] = 740

Ni(L_Ald_-5Me4Im)_2_: 2323329, [NiL_2_H^+^] = 503, [NiL_2_Na^+^] = 525, [NiL_2_K^+^] = 541

Ni(A_Ket_-5Me4Im)_2_: 2285098, [NiL(C_4_H_6_N_2_)^+^ ] = 320 [NiL_2_Na^+^] = 441, [NiL_2_-HNa_2_^+^] = 463 and [NiL_2_K^+^] = 457

Ni(V_Ket_-5Me4Im)_2_: 2285096, [NiL_2_H^+^] = 475 [NiL_2_Na^+^] = 497, [NiL_2_K^+^] = 513 and [Ni_2_L_3_^+^] = 740

Ni(L_Ket_-5Me4Im)_2_: 2323331, [NiL_2_H^+^] = 503, [NiL_2_Na^+^] = 525, [NiL_2_K^+^] = 541 and [Ni_2_L_3_^+^] = 782.

## 4. Conclusions

The reactions of an imidazole 2-carboxaldehyde with the anion of an amino acid, method (a). or an imidazole-2 methanamine with a pyruvate, method (b), both give the ketimine tautomer of an amino acid Schiff base, isolated as their nickel(II) complexes. The isolation of the ketimine in both types of reactions shows that under these conditions the aldimine tautomerizes to the ketimine and suggests that the ketimine is the thermodynamically favored product. The structures of the ketimine from either route are identical. In contrast, the reaction of an imidazole-4-carboxaldehyde (method a) or the reaction of an imidazole-4-methanamine (method b) give exclusively the aldimine and ketimine tautomers, respectively. There is no evidence of an equilibrium under these conditions and no conclusion can be drawn regarding the thermodynamically favored species. The significant reactivity difference between 2- and 4-substituted imidazoles may be explained by favorable charge stabilization of the likely intermediate in the conversion of the aldimine to the ketimine possible for the 2- but not the 4-substituted imidazoles. In addition, there is a strong correlation of chirality of the metal and C_α_ stereogenic centers, with the ΛRR and ΔSS enantiomeric pair favored. This could play a role in the stereospecific generation of amino acids in a ketimine-to-aldimine tautomerization. The aldimine and ketimine tautomers of the 4-imidazoles exhibit a 2 × 2 grid supramolecular structure.

## Figures and Tables

**Table 1:** Selected structural parameters, distances in Å and angles in ^o^, for the aldimine and ketimine forms of (a) R = Me Ni(A_Ald_-5Me4Im)_2_ and Ni(A_Ket_-5Me4Im)_2_; (b) R = iPr Ni(V_Ald_-5Me4Im)_2_ and Ni(V_Ket_-5Me4Im)_2_; and (c) R = iBu Ni(L_Ald_-5Me4Im)_2_ and Ni(L_Ket_-5Me4Im)_2_; (compounds (**b**) and (**c**)) are in the Supplemental Information.

Alanine, R = Me					
Distance	Aldimine	Ketimine	Angle	Aldimine	Ketimine
C_ald_-C_Im_	1.451(6) 1.444(6)	1.490(13) 1.497(3)	C_Im_–C_ald_–N_AA_	116.1(4) 116.6(4)	108.32(19) 107.87(17)
C_ald_-N_AA_	1.263(6) 1.264(6)	1.442(3) 1.451(3)	C_Im_–C_ald_–H	121.9 121.7	110.0 110.1
C_ald_-H	0.9300 0.9300	0.9700 0.9700	C_Im_–C_ald_–H’	xxxxx	110.0 110.1
C_ald_-H’	xxxxxx	0.9700 0.9700	N_AA_–C_ald_–H	121.9 121.7	110.0 110.1
			N_AA_–C_ald_–H’	xxxxx	110.0 110.1
			H–C_ald_–H’	xxxxx	108.4 108.4
N_AA_-C_ald_	1.263(6) 1.264(6)	1.442(3) 1.451(3)	Ni–N_AA_–C_ald_	117.0(3) 117.1(3)	118.26(15) 118.48(12)
N_AA_-C_α_	1.484(7) 1.456(5)	1.278(3) 1.271(3)	Ni–N_AA_–C_α_	116.7(3) 116.5(3)	117.63(15) 117.94(15)
N_AA_-Ni	2.010(4) 2.012(4)	2.0259(17) 2.0212(17)	C_α_–N_AA_–C_ald_	126.3(4) 125.7(4)	124.05(19) 123.42(18)
C_α_-N_AA_	1.484(7) 1.456(5)	1.278(3) 1.271(3)	N_AA_–C_α_–C_β_	110.5(6) 113.6(5)	125.6(2) 126.0(2)
C_α_-C_β_	1.503(9) 1.476(8)	1.463(4) 1.473(3)	N_AA_–C_α_–C_CA_	107.5(5) 108.7(4)	113.88(19) 114.04(19)
C_α_-C_CA_	1.543(9) 1.536(7)	1.538(3) 1.530(3)	N_AA_–C_α_–H	109.0 107.6	xxxxx
C_α_-H	0.9800 0.9800	xxxxxx	C_β_–C_α_–C_CA_	111.7(6) 111.5(5)	120.6(2) 119.95(19)
			C_β_–C_α_–H	109.0 107.6	xxxxx
			C_CA_–C_α_–H	109.0 107.6	xxxxx

**Table 2:** Correlation of Chirality on the Nickel and alpha Carbon Stereogenic Centers of Ni(AA_Ald_–Im)_2_ Complexes.

Complex	Amino Acid Used	CCDC Code or Deposition Number	SG	Chirality of Ni and Alpha C Atom(s) Found in Crystal	Chiral or Racemic
Ni(V_Ald_-5Me4Im)_2_	DL Valine	2206541	C2/c	ΔSS and ɅRR in equal quantity in crystal	Racemic
Ni(L_Ald_-5Me4Im)_2_	DL leucine	2323329	C2/c	ΔSS and ɅRR in equal quantity in crystal	Racemic
Ni(F_Ald_-5Me4Im)_2_	DL Phenylalanine	OFIJOE ZEXHUJ	P2_1_/c	ΔSS and ɅRR in equal quantity in crystal	Racemic
Ni(F_Ald_-5Me4Im)_2_	L Phenylalanine	OFIJIY	P2_1_	ΔSS only	Chiral in bulk due to the source of AA
Ni(F_Ald_-4Im)_2_	DL Phenylalanine	986005	P4_1_2_1_2	ɅRR only in crystal	Homochiral in crystal, separation achieved at the level of crystal. Bulk racemic
Ni(V_Ald_-2Im)_2_	DL valine	961029	P4_1_2_1_2	ɅRR only in crystal	Homochiral in the crystal, separation achieved at the level of crystal. Bulk racemic
Ni(V_Ald_-2Im)_2_	DL Valine	961030	P4_3_2_1_2	ΔSS only in crystal	Homochiral in the crystal, separation achieved at the level of crystal. Bulk racemic
Ni(L_Ket_–Nme2Im) (L_Ald_-NMe2Im)	DL Leucine	NISXOG 967169	P-1	ΔS and ɅR in equal quantity in crystal	Racemic

## Data Availability

Data are contained within the article and Supplementary Materials.

## Supplementary Materials

The following supporting information can be downloaded at: https://www.mdpi.com/article/10.3390/molecules29061324/s1, Table S1: Crystallographic Data for new Nickel Ketimine and Aldimine complexes; Table S2: Selected structural parameters, distances in Å and trans angles in ^o^, involving the nickel(II) for the aldimine and ketimine forms of (a) R = Me Ni(A_Ald_-5Me4Im)_2_ and Ni(A_Ket_-5Me4Im)_2_ (b) R = iPr Ni(V_Ald_-5Me4Im)_2_ and (c) R = iBu Ni(L_Ald_-5Me4Im)_2_ and Ni(L_Ket_-5Me4Im)_2_; Table S3: Selected structural parameters, distances in Å and angles in ^o^, for the aldimine and ketimine forms of (a) R = Me Ni(A_Ald_-5Me4Im)_2_ and Ni(A_Ket_-5Me4Im)_2_ (in text) (b) R = iPr Ni(V_Ald_-5Me4Im)_2_ and Ni(V_Ket_-5Me4Im)_2_ and (c) R = iBu Ni(L_Ald_-5Me4Im)_2_ and Ni(L_Ket_-5Me4Im)_2._
