# Supplementary material for: Selective Generation of Aldimine and Ketimine Tautomers of the Schiff Base Condensates of Amino Acids with Imidazole Aldehydes or of Imidazole Methanamines with Pyruvates—Isomeric Control with 2- vs. 4-Substituted Imidazoles
Source: Molecules. 2024 Mar 16;29(6):1324. doi: 10.3390/molecules29061324 (PMC10974184; doi:10.3390/molecules29061324)
Supplement: Supplementary file 1 [file molecules-29-01324-s001.zip › molecules-2890553-supplementary.pdf]

SUPPLEMENTAL INFORMATION

Selective Generation of Aldimine and Ketimine Tautomers of the Schiff Base Condensates of Amino Acids with Imidazole Aldehydes or of Imidazole Methanamines with Pyruvates—Isomeric Control with 2- vs 4- substituted Imidazoles

Greg Brewer[a], Cynthia Brewer[a], Raymond J. Butcher[b], Peter Zavalij[c]

[a] Department *of Chemistry, The Catholic University of America, Washington, DC 20064, USA*

[b] *Department of Chemistry, Howard University, Washington, DC 20059, USA*

[c] *Department of Chemistry, University of Maryland, College Park, Md 20742, USA*

Table S1 Crystallographic Data for new Nickel Ketimine and Aldimine complexes

Table S2. Selected structural parameters, distances in Å and trans angles in °, involving the nickel(II) for the aldimine and ketimine forms of a) R=Me Ni(A_{Ald}-5Me4Im)$_2$ and Ni(A_{Ket}-5Me4Im)$_2$ b) R=iPr Ni(V_{Ald}-5Me4Im)$_2$ and and c) R=iBu Ni(L_{Ald}-5Me4Im)$_2$ and Ni(L_{Ket}-5Me4Im)$_2$.

Table S3 Selected structural parameters, distances in Å and angles in °, for the aldimine and ketimine forms of a) R=Me Ni(A_{Ald}-5Me4Im)$_2$ and Ni(A_{Ket}-5Me4Im)$_2$ (in text) b) R=iPr Ni(V_{Ald}-5Me4Im)$_2$ and Ni(V_{Ket}-5Me4Im)$_2$ and c) R=iBu Ni(L_{Ald}-5Me4Im)$_2$ and Ni(L_{Ket}-5Me4Im)$_2$(b) and c) in Supplemental.

ORTEP drawing of each complex

IR spectra for each complex

ESI MS for each complex

Table S1 Crystallographic Data for new Nickel Ketimine and Aldimine complexes

Compound	**Ni(A_{Ket}-2Im)$_2$ ·3H_2 O**	**Ni(A_{Ket}-NMe2Im)$_2$ ·2H_2O**	**[Ni(L_{Ket}-NMe2Im)$_2$ ·1.5 H_2O**	**Ni(A_{Ald}-5Me4Im)$_2$ ·H_2O**	**Ni(A_{Ket}-5Me4Im)$_2$ ·2H_2O**
CSD number	2015580	2285095	2323328	2285097	2285098
Empirical formula	$C_{14}H_{16}N_6NiO_4$ ·3H_2O	$C_{16}H_{20}N_6NiO_4$ · 2 H_2O	$C_{44}H_{64}N_{12}Ni_2O_8$ ··3H_2O	$C_{16}H_{20}N_6NiO_4$ · H_2O	$C_{16}H_{20}N_6NiO_4$ ·2H_2O
M/ g mol^{-1}	443.07	455.12	1060.54	437.10	455.12
Temperature /K	150(2)	293(2)	100(2)	293(2)	297(2)
λ/ Å	0.71073	1.54184	1.54184	1.54178	1.54178
Crystal System	Monoclinic	Monoclinic	Triclinic	Monoclinic	Orthorhombic
Space group	P21/n	P21/n	P-1	P21/n	Pbca
Unit cell dimensions	a=11.2052(5) Å, b=15.4495(7) Å, c=11.4742(5) Å, α= 90° β=106.8057(7)° γ= 90°	a=9.3926(2) Å, b=18.7718(3) Å, c=11.6171(2) Å, α= 90° β=105.188(2)° γ= 90°	a=10.6058(6) Å, b=12.2664(7) Å, c=12.4833(7) Å, α= 61.850(6)° β=83.338(5)° γ= 66.723(6)°	a=14.2064(8) Å, b=9.0827(4) Å, c=16.1368(10) Å, α= 90° β=112.662(7)° γ= 90°	a=9.5290(9) Å, b=16.1523(4) Å, c=27.4249(6) Å, α= 90° β=90° γ= 90°
Volume/ Å^3	1901.52(15)	1976.74(7)	1310.39(15)	1921.4(2)	4221.11(17)
Z	4	4	1	4	8
Abs. Coeff./mm^{-1}	1.070	1.837	1.445	1.826	1.720
F(000)	920.0	952	562	912	1904
Crystal size/ mm^3	0.41x0.37x0.35		0.415x0.084x0.066		0.240x0.186x0.170
Theta range/°	4.476 to 62.498	4.594 to 78.875	4.032 to 76.487	3.531 to 75.066	3.223 to 76.260
Index ranges	-16≤h≤16 -22≤k≤22 -16≤l≤16	-11≤h≤11 -22≤k≤23 -13≤l≤14	-13≤h≤13 -7≤k≤15 -15≤l≤15	-16≤h≤17 -10≤k≤11 -20≤l≤18	-12≤h≤10 -20≤k≤20 -33≤l≤34
Reflections Collected	33439	35489	14315	30655	52407
Independent Reflections	6168	4064	5163	3859	4383
R1	0.0365	0.0715	0.0762	0.0726	0.0410
wR2	0.0757	0.2016	0.1930	0.1906	0.1105
GOF on F^2	1.000	1.065	1.069	1.067	1.053

Table S1 cont.

Compound	**Ni(V_{Ald}-5Me4Im)$_2$**	**Ni(V_{Ket}-5Me4Im)$_2$**	**Ni(L_{Ald}-5Me4Im)$_2$**	**Ni(L_{ket}-5Me4Im)$_2$**
CSD number	2206541	2285096	2323329	2323331
Empirical formula	$C_{20}H_{28}N_6NiO_4$	$C_{20}H_{28}N_6NiO_4$	$C_{22}H_{32}N_6NiO_4$	$C_{22}H_{32}N_6NiO_4$
M/ g mol^{-1}	475.19	475.19	503.24	503.24
Temperature/ K	102(2)	263(2)	100(2)	170(2)
λ/ Å	0.71073	1.54178	1.54184	0.71073
Crystal System	Monoclinic	Orthorhombic	Monoclinic	Monoclinic
Space group	C2/c	Pbcn	C2/c	C2/c
Unit cell dimensions	a=25.658(4) Å,<br>b=29.909(4) Å,<br>c=12.8720(19) Å, α= 90°<br>β=110.693(7)°<br>γ= 90°	a=11.59070(10) Å,<br>b=13.91300(10) Å,<br>c=13.92430(10) Å, α= 90°<br>β=90°<br>γ= 90°	a=14.7896(4) Å,<br>b=14.4214(4) Å,<br>c=12.5723(3) Å, α= 90°<br>β=110.891(2)°<br>γ= 90°	a=15.7370(7) Å,<br>b=13.8295(6) Å,<br>c=12.6156(5) Å, α= 90°<br>β=115.467(2)°<br>γ= 90°
Volume/ Å^3	9241(2)	2245.45(3)	2506.75(12)	2478.81(19)
Z	16	4	4	4
Abs. Coeff./mm^{-1}	0.877	1.573	1.438	0.821
F(000)	4000	1000	1064	1064.0
Crystal size/ mm^3		0.287x0.226x0.105	0.245x0.139x0.127	0.19x0.03x0.025
Theta range/°	2.171 to 25.259	4.966 to 76.310	4.430 to 76.217	4.568to 60.204
Index ranges	$-30\leq h\leq 30$<br>$-35\leq k\leq 35$<br>$-15\leq l\leq 15$	$-12\leq h\leq 14$<br>$-17\leq k\leq 17$<br>$-17\leq l\leq 17$	$-18\leq h\leq 18$<br>$-15\leq k\leq 18$<br>$-15\leq l\leq 15$	$-22\leq h\leq 22$<br>$-19\leq k\leq 19$<br>$-17\leq l\leq 17$
Reflections Collected	87793	117086	22899	47084
Independent Reflections	8372	2344	2581	3639
R1	0.0871	0.0325	0.0788	0.0321
wR2	0.1901	0.0900	0.2091	0.0730
GOF on F^2	1.065	1.051	1.127	1.052

Table S2. Selected structural parameters, distances in Å and trans angles in °, involving the nickel(II) for the aldimine and ketimine forms of a) R=Me Ni(A_{Ald}-5Me4Im)$_2$ and Ni(A_{Ket}-5Me4Im)$_2$ b) R=iPr Ni(V_{Ald}-5Me4Im)$_2$ and and c) R=iBu Ni(L_{Ald}-5Me4Im)$_2$ and Ni(L_{Ket}-5Me4Im)$_2$.

Parameter\cpd.	Ni(A_{Ald}-5Me4Im)$_2$	Ni(A_{Ket}-5Me4Im)$_2$	Ni(V_{Ald}-5Me4Im)$_2$	Ni(V_{Ket}-5Me4Im)$_2$	Ni(L_{Ald}-5Me4Im)$_2$	Ni(L_{Ket}-5Me4Im)$_2$
Ni-N_{Im}	2.089(4)<br>2.087(4)	2.0550(19)<br>2.0563(18)	2.106(6)<br>2.117(6)	2.0628(14)<br>2.0628(13)	2.095(2)	2.0663(11)
Ni-N_{AA}	2.010(4)<br>2.012(4)	2.0259(17)<br>2.0212(17)	2.028(5)<br>2.012(5)	2.0288(12)<br>2.0288(12)	2.024(3)	2.0221(10)
Ni-O	2.074(13)<br>2.088(4)	2.1105(18)<br>2.1109(16)	2.080(5)<br>2.069(5)	2.0848(11)<br>2.0848(11)	2.077(2)	2.0974(10)
N_{AA}-Ni-N_{AA}	174.38(17)	178.60(7)	170.1(3)	177.58(7)	177.83(14)	178.57(7)
N_{Im}-Ni-O	159.60(13)<br>159.8(3)	157.34(7)<br>157.14(7)	158.9(2)<br>158.8(2)	158.86(5)<br>158.87(5)	158.31(9)<br>158.31(9)	157.97(4)<br>157.97(4)

Table S3 Selected structural parameters, distances in Å and angles in °, for the aldimine and ketimine forms of a) R=Me $Ni(A_{Ald}\text{-}5Me4Im)_2$ and $Ni(A_{Ket}\text{-}5Me4Im)_2$ (in text) b) R=iPr $Ni(V_{Ald}\text{-}5Me4Im)_2$ and $Ni(V_{Ket}\text{-}5Me4Im)_2$ and c) R=iBu $Ni(L_{Ald}\text{-}5Me4Im)_2$ and $Ni(L_{Ket}\text{-}5Me4Im)_2$ (in supplemental).

b) Valine, R= i-Pr

Distance	Aldimine	Ketimine	Angle	Aldimine	Ketimine
C_{Ald}-C_{Im}	1.451(9)<br>1.432(10)	1.495(2)	C_{Im} –C_{Ald} –N_{AA}	116.0(6)<br>116.1(6)	108.56(12)
C_{Ald}-N_{AA}	1.275(9)<br>1.276(9)	1.4645(19)	C_{Im} –C_{Ald} –H	122.0<br>121.9	110.0
C_{Ald}-H	0.9500<br>0.9500	0.9700	C_{Im} –C_{Ald} –H’	xxxx	110.0
C_{Ald}-H’	xxxxxx	0.9700	N_{AA} –C_{Ald} –H	122.0<br>121.9	110.0
			N_{AA} –C_{Ald} –H’	xxxxx	110.0
			H –C_{Ald} –H’	xxxxx	108.4
N_{AA}-C_{Ald}	1.275(9)<br>1.276(9)	1.4645(19)	Ni–N_{AA}–C_{Ald}	116.5(4)<br>116.9(5)	116.89(9)
N_{AA}-C_α	1.454(8)<br>1.48(2)	1.2683(19)	Ni–N_{AA}–C_α	117.0(4)<br>115.8(8)	117.53(10)
N_{AA}-Ni	2.028(5)<br>2.012(5)	2.0288(12)	C_α–N_{AA}–C_{Ald}	124.5(6)<br>126.9(9)	124.81(13)
C_α-N_{AA}	1.454(8)<br>1.48(2)	1.2683(19)	N_{AA}–C_α–C_β	113.1(6)<br>114.9(15)	125.03(13)
C_α-C_β	1.555(9)<br>1.552(11)	1.516(2)	N_{AA}–C_α–C_{CA}	107.5(9)<br>108.9(10)	113.01(13)
C_α-C_{CA}	1.536(10)<br>1.48(2)	1.554(2)	N_{AA}–C_α–H	108.6<br>107.7	xxxxx
C_α-H	1.000<br>1.000	xxxxxx	C_β–C_α–C_{CA}	110.0(6)<br>109.7(15)	121.94(15)
			C_β–C_α–H	108.6<br>107.7	xxxxx
			C_{CA}–C_α–H	108.6<br>107.7	xxxxx

c) Leucine, R=iBu

Distance	Aldimine	Ketimine	Angle	Aldimine	Ketimine
C_{Ald}-C_{Im}	1.450(4)	1.4966(19)	C_{Im} –C_{Ald} –N_{AA}	115.6(3)	107.39(11)
C_{Ald}-N_{AA}	1.280(4)	1.4638(16)	C_{Im} –C_{Ald} –H	122.2	110
C_{Ald}-H	0.9500	0.990	C_{Im} –C_{Ald} –H’	xxxxx	110
C_{Ald}-H’	xxxxxx	0.990	N_{AA} –C_{Ald} –H	122.2	110
			N_{AA} –C_{Ald} –H’	xxxxx	110
			H –C_{Ald} –H’	xxxxx	109
N_{AA}-C_{Ald}	1.280(4)	1.4638(16)	Ni–N_{AA}–C_{Ald}	117.2(2)	118.41(8)
N_{AA}-C_{α}	1.458(4)	1.2732(16)	Ni–N_{AA}–C_{α}	117.36(19)	118.05(9)
N_{AA}-Ni	2.024(3)	2.0221(10)	C_{α}–N_{AA}–C_{Ald}	124.9(3)	123.52(11)
C_{α}-N_{AA}	1.458(4)	1.2732(16)	N_{AA}–C_{α}–C_{β}	112.1(3)	126.68(12)
C_{α}-C_{β}	1.516(4)	1.4989(19)	N_{AA}–C_{α}–C_{CA}	107.1(2)	113.07(12)
C_{α}-C_{CA}	1.553(4)	1.5453(18)	N_{AA}–C_{α}–H	109.9	xxxxx
C_{α}-H	1.000	xxxxxx	C_{β}–C_{α}–C_{CA}	107.8(3)	120.25(12)
			C_{β}–C_{α}–H	109.9	xxxxx
			C_{CA}–C_{α}–H	109.9	xxxxx
